# Analysis of tweets on toothache during the COVID-19 pandemic using the CrystalFeel algorithm: a cross-sectional study

**DOI:** 10.1186/s12903-021-01778-8

**Published:** 2021-08-25

**Authors:** Halenur Altan, Alem Coşgun

**Affiliations:** Department of Pediatric Dentistry, Faculty of Dentistry, Tokat Gaziosmanpaşa University, Tokat, Turkey

**Keywords:** COVID-19, Pandemic, Emotions, Social media, Toothache

## Abstract

**Background:**

Reasons such as the lack of information on the COVID-19 disease, lack of proven treatment for it, uncertainty about the duration of the pandemic, or social isolation affect people’s mental health. This study aimed to analyze the emotional type and intensity in tweets on toothache posted during the COVID-19 pandemic.

**Methods:**

Using the Twitter Search API, we collected tweets in English associated with the keywords “Corona, Toothache” “Corona, Tooth, Pain” “Corona, Dental Pain” “Covid-19, Toothache” “Covid-19, Tooth, Pain” and “Covid-19, Dental Pain” posted between March 11, 2020 and June 30, 2020 all around the world. After the relevant inclusion and exclusion criteria were applied, 426 posts were selected and analyzed using the CrystalFeel algorithm, a sensitivity analytical technology with proven accuracy. The chi-square test (SPSS v23, IBM) was used to compare emotions and emotional intensities according to the words used.

**Results:**

It was determined that 80.3% of the participants experienced fear and 61.7% had a negative emotional intensity. There was no statistically significant difference between the distributions of emotions according to the words without time distinction (p = 0.136). There was a statistically significant difference between the distributions of emotional intensity according to the words without time distinction (p = 0.006). The keyword “Corona, Toothache” was used the most frequently by 30.8% of the participants.

**Conclusions:**

This study is the first to analyze the emotional reactions of individuals who experienced toothaches during the COVID-19 pandemic using the CrystalFeel algorithm. Monitoring the social media posts of individuals experiencing toothache during the pandemic will help reduce fear and anger emotions and design public information messages that are compatible with the target group’s needs.

## Background

The World Health Organization (WHO) announced on March 11, 2020 that the outbreak of a new type of coronavirus (COVID-19) constituted an alarming international public health emergency and declared it a global pandemic [1]. The outbreak, which spread to the whole world and turned into a pandemic in a short amount of time, became of particular concern to dentists and the patient group in need of dental treatment due to the characteristics of its transmission routes. Working close to the patient and the aerosols and droplets generated during the treatment constitute novel and significant risks for dentists regarding highly infectious epidemics, such as COVID-19 [2, 3]. During the pandemic, while some dental institutions and clinics completely stopped the admission of patients, some of them continued to provide only emergency treatment and care services [4].

Pain is a difficult experience and is generally the most important condition requiring health support [5]. Severe toothache, dentoalveolar trauma, and swelling due to abscess are the main emergency dental interventions, and pain control should be provided [6]. Untreated pain has physical effects, such as difficulty breathing, increased heart rate and blood pressure, myocardial oxygen requirement, and increased stress hormones, vomiting, and muscle tension [7]. Toothache can affect social well-being and negatively impact the individual’s quality of life [8]. The frequency and severity of dental pain negatively affect daily nutrition, sleep, and smiling and oral care [9, 10]. In addition to physical effects, pain may also increase emotions such as anxiety, fear, distress, helplessness, and hopelessness [11]. Furthermore, the management of this condition imposes a huge financial burden on society and individuals [12, 13].

Social media sites (Facebook, Instagram, and Twitter) allow users to play active roles in reporting and disseminating news and sharing insights and apprehensions while disseminating interpretations of health events outside a public health context [14]. Twitter is a well-known microblogging service where users post messages, denoted “tweets”. These are short messages with a maximum length of (currently) 280 characters. Since people who post those messages share preferences and opinions with other users, tweets are a valuable source of people’s opinions and sentiments [15]. Over 645 million registered Twitter users exist globally to distribute over 58 million tweets daily [16]. The community of Twitter users reflects a diverse and rapidly growing global population [17].

In the past decade, social media analytic tools have been utilized to monitor public sentiments and communication patterns of public health emergencies like the Ebola and Zika epidemics [18]. The analysis of the emotional responses of individuals who experienced toothache during the COVID-19 pandemic through texts shared on social media may provide a quick understanding of the needs in health-related planning. In this study, the authors aimed to analyze the number and content of tweets on toothache during the peak of the COVID-19 pandemic by using the CrystalFeel algorithm, which is a sensitivity analytical artificial intelligence technology.

## Methods

### Study design

Considering the intensity distributions; The number of samples to be included in the study was determined as 420 with 95% confidence (1-α), 80% test power (1-β) and w = 0.1368 effect size [19].

Tweets in English posted between 11 and 2020 and 30 June 2020 worldwide were included in the study. Authors analyzed the data considering those periods: 11 March 2020–31 March 2020; 1 April 2020–3 April 02020; 1 May 2020–31 May 2020; and 1 June 2020–30 June 2020, because the World Health Organization (WHO) declared COVID-19 a global epidemic on March 11, 2020. Popular tweets were found using the keywords “Corona, Toothache” “Corona, Tooth, Pain” “Corona, Dental Pain” “Covid-19, Toothache” “Covid-19, Tooth, Pain” and “Covid-19, Dental Pain” in the Twitter Search API. These tweets represented the emotions of individuals who experienced toothache after the COVID-19 pandemic started.

Two researchers (A.C and H.A) collected the tweet texts regarding its inclusion and exclusion criteria, and the posts were analyzed using CrystalFeel Algorithm. The researchers checked the data obtained using the CrystalFeel Algorithm. Account of Twitter was used only to collect information (with no followers). Only the tweet texts were collected, excluding age, gender, and user type. Exclusion criteria included the following: advertising or promotional posts, posts involving statements that did not include content that could be used to make inferences about how people felt about their braces, hyperlinks (content beginning with “http://” or “https://”), posts that had been posted previously and then re-posted by another user (retweets), irrelevant, incoherent or ambiguous posts, non-English posts, and tweets sent from corporate accounts—since these were posted for informational purposes and had no emotional meaning. Metaphorical sentences containing allegory were also not included in the study. Tweets containing multiple keywords were used only once. Furthermore, tweets containing only emojis, icons, and videos were excluded from the study.

No ethics committee approval was required for this work since only public data were used and no human subjects were included.

### Analysis of emotions

The emotions underlying the tweets were analyzed using the CrystalFeel algorithm, which is a proven sensitivity analytical technology. CrystalFeel Algorithm gives us five emotion dimensions and five emotion intensities. Also, this program reports the most prominent emotion. In this study, the most prominent emotion and related emotion intensity from analyzed tweets chose only by researchers.

CrystalFeel is a collection of five machine-learning-based algorithms that run simultaneously and independently to analyze the emotional characteristics of a text, providing five output dimensions: anger intensity, fear intensity, sadness intensity, joy intensity, and valence intensity. The intensities were expressed as continuous values between 0 and 1. 0—this text expresses no emotional intensity or at a very low level; 1—this text expresses extremely high emotional intensity. Besides, based on the intensity scores, CrystalFeel Algorithm also generates two converted outputs: sentiment category and emotion category [20].

According to Plutchik’s wheel of emotions, sadness-joy and anger-fear are the basic emotional pairs of opposite experiences [21]. Sadness is an unpleasant emotion caused by something that didn’t happen or something that wasn’t wanted to happen, while Joy is a positive emotion that occurs when something is highly desired or pleasant [22]. Fear is an unpleasant feeling caused by the thought of danger, while anger is caused by uncertainty caused by others [22]. Investigating the evolution of these four basic emotions may show the changing dynamics of public experience in crisis [23] (Table 1).

**Table 1:** Examples of the tweets data and their corresponding emotion and emotion intensity classification results

Tweet	Emotion	Emotion intensity
The pain from my tooth is making my throat hurt or i’m just allergic to my medication OR i have corona. i’m gonna play tlou until something terrible happens to me stay tuned	Fear	Very negative
I may not die of COVID-19 but I might die of pain because no dental clinic is open nearby and there’s no way I could have my rebellious premolar extracted anytime soon.	Fear	Very negative
I have such a bad toothache and my dentist is closed because of Corona	Sadness	Very negative
Lol corona makes the toothache worse I can’t dzeel	Sadness	Negative
I have chronic TMJ problems which under the new COVID-19 VIC legislation I can’t get treatment for. I’ve now cracked a tooth causing terrible pain and need a crown. I also can’t get treatment for that. sort out what is deemed “essential services”.	Anger	Negative
Just a bunch of toothache and corona pisses me off	Anger	Very negative
I’ve been locked where I live for a week since I’m not allowed to leave the house because of “Corona”, when I’ve had a much intense toothache in several of my teeth for the whole week and I’ve needed a doctor. Apparently I can only leave the house “when the government allows it”.	Anger	Negative
Thank you Dr. Ayushi Mishra,thank you moolchand. During these tough times of Corona virus lockdown she suggested me few medicines for my intolerable wisdom tooth pain. Greatly relieved.Thanks again	Joy	Positive

### Statistical analysis

Data were analyzed with IBM SPSS V23. Chi-square test was used to compare emotions and emotion intensities according to the words used. The comparison of the ratios of each column as a result of the chi-square test was performed with the Bonferroni test. Analysis results were presented as frequency (percentage) for categorical data. Significance level was taken as p < 0.050.

## Results

In total, 842 tweets were obtained by entering the specified keywords in the search application of Twitter. After applying the exclusion criteria, 416 tweets were removed. The remaining 426 tweets were included in the study and analyzed.

It was determined that 80.3% of the participants experienced fear and that 61.7% had a negative emotional intensity; 38% of the keywords were used mostly between 1 and 2020 and 30 June 2020. The keyword “Corona, Toothache” was used the most frequently by 30.8% of the participants (Table 2).

**Table 2:** Descriptive statistics of the variables

	Frequency (n)	Percent (%)
Emotions		
Fear	342	80.3
Anger	50	11.7
Sadness	25	5.9
Joy	9	2.1
Intensity		
Negative	263	61.7
Very negative	154	36.2
Positive	9	2.1
Date		
1 April 2020–30 April 2020	162	38.0
11 March 2020–31 March 2020	135	31.7
1 May 2020–31 May 2020	96	22.5
1 June 2020–30 June 2020	33	7.7
Group		
“Corona, Toothache”	131	30.8
“Covid-19, Toothache”	99	23.2
“Covid-19, Dental Pain”	64	15.0
“Covid-19,Tooth, Pain”	56	13.1
“Corona, Tooth, Pain”	53	12.4
“Corona, Dental Pain”	23	5.4

There was no statistically significant difference between the distributions of emotions according to the words used between the following periods: 11 March 2020–31 March 2020 (p = 0.510); 1 April 2020–3 April 02020 (p = 0.410); 1 May 2020–31 May 2020 (p = 0.328); and 1 June 2020–30 June 2020 (p = 0.074). There was also no statistically significant difference between the distributions of emotions according to the words without time distinction (p = 0.136) (Table 3).

**Table 3:** Comparison of emotions according to the words used on different dates

Date	Emotions	“Corona, Dental Pain”	“Corona, Tooth, Pain”	“Corona, Toothache”	“Covid-19, Dental Pain”	“Covid-19, Tooth, Pain”	“Covid-19, Toothache”	Test statistics	p
11 March 2020–31 March 2020	Joy	0 (0)	1 (7,7)	0 (0)	1 (4,5)	0 (0)	0 (0)	${\chi }^{2}$ = 14,207	0.510
	Anger	1 (14,3)	4 (30,8)	10 (20)	1 (4,5)	2 (15,4)	5 (16,7)		
	Fear	6 (85,7)	8 (61,5)	39 (78)	19 (86,4)	10 (76,9)	25 (83,3)		
	Sadness	0 (0)	0 (0)	1 (2)	1 (4,5)	1 (7,7)	0 (0)		
	Total	7 (100)	13 (100)	50 (100)	22 (100)	13 (100)	30 (100)		
1 April 2020–30 April 2020	Joy	0 (0)	0 (0)	0 (0)	1 (5)	1 (3,8)	3 (10)	${\chi }^{2}$ = 15,581	0.410
	Anger	0 (0)	1 (5,3)	8 (14,8)	1 (5)	3 (11,5)	2 (6,7)		
	Fear	13 (100)	18 (94,7)	42 (77,8)	17 (85)	20 (76,9)	24 (80)		
	Sadness	0 (0)	0 (0)	4 (7,4)	1 (5)	2 (7,7)	1 (3,3)		
	Total	13 (100)	19 (100)	54 (100)	20 (100)	26 (100)	30 (100)		
1 May 2020–31 May 2020	Joy	0 (0)	1 (6,3)	0 (0)	0 (0)	0 (0)	1 (3,1)	${\chi }^{2}$ = 16,850	0.328
	Anger	1 (100)	1 (6,3)	2 (11,1)	1 (5,6)	1 (9,1)	2 (6,3)		
	Fear	0 (0)	12 (75)	15 (83,3)	13 (72,2)	9 (81,8)	26 (81,3)		
	Sadness	0 (0)	2 (12,5)	1 (5,6)	4 (22,2)	1 (9,1)	3 (9,4)		
	Total	1 (100)	16 (100)	18 (100)	18 (100)	11 (100)	32 (100)		
1 June 2020–30 June 2020	Anger	0 (0)	1 (20)	2 (22,2)	0 (0)	0 (0)	1 (14,3)	${\chi }^{2}$ = 17,014	0.074
	Fear	2 (100)	4 (80)	7 (77,8)	4 (100)	3 (50)	6 (85,7)		
	Sadness	0 (0)	0 (0)	0 (0)	0 (0)	3 (50)	0 (0)		
	Total	2 (100)	5 (100)	9 (100)	4 (100)	6 (100)	7 (100)		
Total	Joy	0 (0)	2 (3,8)	0 (0)	2 (3,1)	1 (1,8)	4 (4)	${\chi }^{2}$ = 21,037	0.136
	Anger	2 (8,7)	7 (13,2)	22 (16,8)	3 (4,7)	6 (10,7)	10 (10,1)		
	Fear	21 (91,3)	42 (79,2)	103 (78,6)	53 (82,8)	42 (75)	81 (81,8)		
	Sadness	0 (0)	2 (3,8)	6 (4,6)	6 (9,4)	7 (12,5)	4 (4)		
	Total	23 (100)	53 (100)	131 (100)	64 (100)	56 (100)	99 (100)		

${\chi }^{2}$: Chi-square test statistics

However, there was a statistically significant difference between the distributions of emotional intensity according to the words used between 11 and 2020 and 31 March 2020 (p = 0.006). There was a statistically significant difference between the distributions of emotional intensity according to the words without time distinction (p = 0.006). Finally, there was no statistically significant difference between the distributions of emotional intensity according to the words used between the following periods: 1 April 2020–30 April 2020 (p = 0.141); 1 May 2020–31 May 2020 (p = 0.709); and 1 June 2020–30 June 2020 (p = 0.093) (Table 4).

**Table 4:** Comparison of emotional intensity according to the words used on different dates

Date	Intensity	“Corona, Dental Pain”	“Corona, Tooth, Pain”	“Corona, Toothache”	“Covid-19, Dental Pain”	“Covid-19, Tooth, Pain”	“Covid-19, Toothache”	Test statistics	p
11 March 2020–31 March 2020	Very negative	4 (57,1)^a^	6 (46,2)^ab^	21 (42)^ab^	5 (22,7)^ab^	0 (0)^b^	4 (13,3)^ab^	${\chi }^{2}$ = 24,498	**0.006**
	Negative	3 (42,9)^a^	6 (46,2)^a^	29 (58)^ab^	16 (72,7)^ab^	13 (100)^b^	26 (86,7)^ab^		
	Positive	0 (0)	1 (7,7)	0 (0)	1 (4,5)	0 (0)	0 (0)		
	Total	7 (100)	13 (100)	50 (100)	22 (100)	13 (100)	30 (100)		
1 April 2020–30 April 2020	Very negative	6 (46,2)	13 (68,4)	23 (42,6)	9 (45)	11 (42,3)	8 (26,7)	${\chi }^{2}$ = 14,771	0.141
	Negative	7 (53,8)	6 (31,6)	31 (57,4)	10 (50)	14 (53,8)	19 (63,3)		
	Positive	0 (0)	0 (0)	0 (0)	1 (5)	1 (3,8)	3 (10)		
	Total	13 (100)	19 (100)	54 (100)	20 (100)	26 (100)	30 (100)		
1 May 2020– 31 May 2020	Very negative	1 (100)	7 (43,8)	6 (33,3)	6 (33,3)	2 (18,2)	9 (28,1)	${\chi }^{2}$ = 7,177	0.709
	Negative	0 (0)	8 (50)	12 (66,7)	12 (66,7)	9 (81,8)	22 (68,8)		
	Positive	0 (0)	1 (6,3)	0 (0)	0 (0)	0 (0)	1 (3,1)		
	Total	1 (100)	16 (100)	18 (100)	18 (100)	11 (100)	32 (100)		
1 June 2020–30 June 2020	Very negative	0 (0)	3 (60)	3 (33,3)	2 (50)	0 (0)	5 (71,4)	${\chi }^{2}$ = 9,425	0.093
	Negative	2 (100)	2 (40)	6 (66,7)	2 (50)	6 (100)	2 (28,6)		
	Total	2 (100)	5 (100)	9 (100)	4 (100)	6 (100)	7 (100)		
Total	Very negative	11 (47,8)^ab^	29 (54,7)^b^	53 (40,5)^ab^	22 (34,4)^ab^	13 (23,2)^a^	26 (26,3)^a^	${\chi }^{2}$ = 24,518	**0.006**
	Negative	12 (52,2)^ab^	22 (41,5)^b^	78 (59,5)^ab^	40 (62,5)^ab^	42 (75)^a^	69 (69,7)^a^		
	Positive	0 (0)	2 (3,8)	0 (0)	2 (3,1)	1 (1,8)	4 (4)		
	Total	23 (100)	53 (100)	131 (100)	64 (100)	56 (100)	99 (100)		

${\chi }^{2}$: Chi-square test statist; ^a–b^ same letters indicate no statistical difference

## Discussion

During the COVID-19 pandemic, most dental treatments had to be delayed or canceled for the safety of patients and healthcare workers [24]. The pandemic caused changes in people’s moods until it was brought under control, and we could quickly learn about these changes through social media. Due to easy access to social networks such as Twitter, researchers can use these tools as sources of data on many topics, such as attitudes toward health-related issues [25, 26]. Twitter analysis revealed an increase in the number of tweets mentioning the toothache during the most intense period of the COVID-19 pandemic (11 March 2020 and 30 June 2020) and the intensity of emotions of “fear” and “anger”.

Toothache disrupts the mood, sleep, eating habits, and social functioning of the individual, negatively affects quality of life, and is a condition that needs to be treated [10, 13, 27]. A study that analyzed the dental treatment needs of Brazilian Twitter users between 23 and 2020 and 4 May 2020 found that the need for treatment due to dental pain was ranked first [28]. Moreover, in the study in which Zhuo-Ying Tao et al. analyzed the nature and spread of oral health information on COVID-19 using the Weibo media application, it was reported that the main health problem requiring treatment was toothache [29]. In this study conducted with different keywords related to toothache, the highest number of tweets were found for the following keywords: “Corona-Toothache” (30.8%), “Covid 19-Toothache” (23.2%), and “Covid-19-Dental Pain” (15.0%), a result in agreement with the literature.

In this study, the emotions underlying the tweets on toothache during the COVID-19 pandemic were analyzed for the first time using the CrystalFeel algorithm, a sensitivity analytical technology. Since tweets in English posted not only in a single region but all over the world were analyzed, the data contributed to obtaining general information about social media users’ toothaches and emotional states.

Except for a few studies, there have not been many investigations of the psychological effects of the COVID-19 pandemic on society [23, 30–32]. In a study conducted by Wang et al., the psychological effects, depression, stress, and anxiety were evaluated in 1210 participants from 194 cities in China at the beginning of the pandemic [33]. The results of the study indicated that 53.8% of these individuals experienced severe psychological effects. Furthermore, 16.5%, 28.8%, and 8.1% of the participants reported moderate to severe depression, anxiety, and stress levels, respectively. In a recent study, it was argued that the COVID-19 pandemic affected orthodontic appointments and provoked patients’ anxiety and that the biggest concern of more than half of the patients was the delay in treatment [26]. The emotions felt about toothache during the pandemic were fear (80.3%), anger (11.7%), sadness (5.9%), and joy (2.1%), respectively. In our study, the feelings of fear and anger were found to be high in March and April, which was considered to be caused by the cancelation of elective and aerosol dental treatments, the inability to receive dental treatment opinions face to face, misinformation, and uncertainty.

In this study, the least number of tweets were related to “joy” and were about medication or dental treatment during the pandemic period. From the beginning of the pandemic until June, the emotional intensity of the tweets on pain showed a negative or very negative distribution. However, in May and June, the number of negative and very negative tweets decreased. It was considered this change to be related to partial control of pandemic and resumption of dental services.

In this study, we found that Twitter users reported high intensity of pain in 89.0% of the tweets (n = 379) by using the bilateral pain intensity variable. In the studies on toothache intensity on social media conducted before the pandemic, pain intensity was high, but the percentage rates were very low compared to our study [34]. In the literature, people’s sharing of their pain experiences on social media is considered one coping method [17, 35]. The authors assume that there was an increase in the number of tweets expressing high pain intensity during the pandemic because people could not receive dental treatment and thus used Twitter to cope with pain and seek support.

The WHO indicated that social media can be used “to attract public attention during an emergency, to facilitate one-to-one communication, to create situational awareness, to monitor and respond to rumors, public reactions and concerns, and to facilitate local level response” [36]. As indicated by the WHO, social media can be used as a very effective tool not only to disseminate information but also to collect feedback. The reactions, comments, and posts of those who read the post can be seen and evaluated instantly, ensuring that their needs are understood and a faster response is developed [37, 38].

The authors believe that Twitter could be used as a source of data in studies to developed community-oriented communication strategies for toothache experiences during and after the pandemic. With such content analysis studies, in cooperation with dental professionals and the government, live broadcasts, “question-answer hour,” and informative videos about oral and dental health can help to guide individuals have toothache during epidemic periods. In this way, increasing negative emotions can be helped to control.

Concerning the limitations of our study, the CrystalFeel program can only analyze emotions in English content; therefore, tweets in other languages were not included in this study. CrystalFeel is also unable to determine emotions conveyed by metaphorical sentences, which prevents the analysis of sentences containing allegory. Another shortcoming of this work consists of having collected data from only one social media platform. This study findings may not be generalizable to offline population. Therefore, caution is advised before assuming the generalizability of the results, as everyone in the population does not use Twitter [39].

## Conclusions

Monitoring the social media posts of individuals experiencing toothache during the pandemic will help reduce fear and anger emotions and design public information messages that are compatible with the target group’s needs.

## Data Availability

The datasets used and/or analyzed during the current study are available from the corresponding author on reasonable request.
